# Impaired Limb Shortening following Stroke: What’s in a Name?

**DOI:** 10.1371/journal.pone.0110140

**Published:** 2014-10-16

**Authors:** Virginia L. Little, Theresa E. McGuirk, Carolynn Patten

**Affiliations:** 1 Department of Physical Therapy, University of Florida, Gainesville, FL, United States of America; 2 Rehabilitation Science Doctoral Program, University of Florida, Gainesville, FL, United States of America; 3 Brain Rehabilitation Research & Development Center, Malcolm-Randall VA Medical Center, Gainesville, FL, United States of America; 4 Department of Applied Physiology and Kinesiology, University of Florida, Gainesville, FL, United States of America; Purdue University, United States of America

## Abstract

**Background:**

Difficulty advancing the paretic limb during the swing phase of gait is a prominent manifestation of walking dysfunction following stroke. This clinically observable sign, frequently referred to as ‘foot drop’, ostensibly results from dorsiflexor weakness.

**Objective:**

Here we investigated the extent to which hip, knee, and ankle motions contribute to impaired paretic limb advancement. We hypothesized that neither: 1) minimal toe clearance and maximal limb shortening during swing nor, 2) the pattern of multiple joint contributions to toe clearance and limb shortening would differ between post-stroke and non-disabled control groups.

**Methods:**

We studied 16 individuals post-stroke during overground walking at self-selected speed and nine non-disabled controls who walked at matched speeds using 3D motion analysis.

**Results:**

No differences were detected with respect to the ankle dorsiflexion contribution to toe clearance post-stroke. Rather, hip flexion had a greater relative influence, while the knee flexion influence on producing toe clearance was reduced.

**Conclusions:**

Similarity in the ankle dorsiflexion, but differences in the hip and knee, contributions to toe clearance between groups argues strongly against dorsiflexion dysfunction as the fundamental impairment of limb advancement post-stroke. Marked reversal in the roles of hip and knee flexion indicates disruption of inter-joint coordination, which most likely results from impairment of the dynamic contribution to knee flexion by the gastrocnemius muscle in preparation for swing. These findings suggest the need to reconsider the notion of foot drop in persons post-stroke. Redirecting the focus of rehabilitation and restoration of hemiparetic walking dysfunction appropriately, towards contributory neuromechanical impairments, will improve outcomes and reduce disability.

## Introduction

Recovery of walking function is among the foremost goals of stroke survivors. [1] Thus, identification of effective and efficient approaches to restore gait post-stroke is a high priority. [1] Current therapies do not sufficiently retrain walking capacity. Nearly half of stroke survivors fail to respond to contemporary treatments intended to remediate gait dysfunction. [2], [3] This lack of therapeutic efficacy results, in part, because the capacity for such recovery is poorly understood. Equally important, current therapies likely fail to address the most appropriate targets for rehabilitation. In this regard, we note an emphasis in current clinical practice and research efforts to remediate and/or compensate for so-called ‘foot drop’. [4]–[6] Implicit in this approach is the assumption of impaired dorsiflexor function. As a result, multiple intervention approaches currently used (e.g., functional electrical stimulation (FES), [7] ankle foot orthoses (AFO), [8] robotic devices, [9] brain-computer interface (BCI) [6]) specifically target dorsiflexor dysfunction.

Paradoxically, there is little evidence to support the presence of dorsiflexor impairment post-stroke. [10]–[14] It is particularly noteworthy that FES applied to the dorsiflexors has been shown to decrease both peak knee flexion in swing and plantarflexion at toe-off, thus exaggerating two well recognized impairments pathognomonic of gait dysfunction post-stroke. [7] This finding contradicts any rationale for targeting dorsiflexion to remediate stroke-related gait dysfunction.

Impaired paretic limb advancement is an obvious and readily observable manifestation of gait dysfunction post-stroke. [15], [16] However, clinical observation and observational gait analysis methods [16] provide limited information regarding the specific neurological and biomechanical impairments that contribute to hemiparetic gait dysfunction. In contrast, biomechanical analysis yields quantitative evaluation to accurately identify: prominent impairments, causal mechanisms of gait dysfunction and potential intervention targets to restore walking function post-stroke. [17], [18].

Limb advancement involves both anterior translation and shortening of the limb to effect forward progression and clearance during swing phase. Limb clearance typically entails simultaneous contributions from all joints of the swing limb. Dorsiflexion alone is not sufficient to clear the ground if the knee is fully extended, thus attributing impaired paretic limb advancement solely to a clearance deficit resulting from dorsiflexor dysfunction ignores other important contributing factors. Classically defined foot drop results from a focal insult to the peroneal nerve while hemiparesis following stroke involves more widespread effects. [19]–[24] Furthermore, limb clearance involves vertical shortening of the swing limb relative to the stance limb with simultaneous contributions from the hip, knee and ankle. Following stroke each of these joint excursions is reduced in the paretic limb. [10] However, as illustrated by the current emphasis on management of ankle dorsiflexion dysfunction, the influence of these reduced individual joint angular excursions on limb clearance and shortening during paretic limb advancement remains poorly understood.

To investigate this phenomenon more comprehensively, we sought an analysis that would enable understanding of the dynamic interactions between swing-limb joints. Here we analyzed hip, knee, and ankle kinematics during walking to determine their relative contributions to limb clearance and limb shortening. We hypothesized: 1) minimal limb clearance and maximal limb shortening would not differ between controls and individuals post-stroke, and 2) the pattern of multiple joint contributions (hip flexion, knee flexion, and ankle dorsiflexion) to limb clearance and limb shortening would not differ between groups.

## Methods

### Participants

We studied 16 individuals with chronic, post-stroke hemiparesis, able to walk independently at least ten meters with an AFO or assistive device, and nine healthy, non-disabled adults (age: 43.67±11.24 yrs; 5 male; height: 1.76±0.09 m; mass: 80.90±19.91 kg). All participants post-stroke (age: 57±14.37 yrs; 13 male; height: 1.76±0.07 m; mass: 85.54±16.19 kg; chronicity: 4.21±1.93 yrs) experienced a single, mono-hemispheric stroke (confirmed with neuroimaging) and revealed hemiparesis, lower extremity (LE) motor dysfunction (LE Fugl Meyer Synergy Score: 15/22±2.78) and gait impairment (SSWS: 0.54±0.26 m/s).

### Ethics Statement

All procedures described herein were approved by the Stanford University Institutional Review Board and conducted according to the principles expressed in the Declaration of Helsinki. All participants provided written informed consent prior to participation.

### Data Collection and Processing

Participants post-stroke were studied while walking overground at their self-selected speed (SSWS) without an AFO or assistive device. Healthy, non-disabled participants walked overground at their SSWS and up to three slower speeds. All participants wore their own footwear, typically a flat, athletic style shoe. Three-dimensional marker data were obtained and labeled using a seven-camera motion capture system (Qualisys AB., Gothenburg, Sweden, 100 Hz) and a modified Cleveland Clinic marker set (five clusters and 23 additional markers) as described by Chen & Patten. [25] Data were modeled in Visual 3D Basic (v 3.99.25.7, C-Motion, Germantown, MD) and processed with custom Matlab (MathWorks Version 7.7.0 R2008b, Natick, MA) scripts. Kinematics were calculated from marker data, filtered (lowpass 4^th^ order Butterworth, 6 Hz cutoff) and time-normalized to the gait cycle. Control data most closely matching the gait speed of individual stroke participants were selected for comparison.

### Gait Events of Interest

Toe clearance served as our proxy for limb clearance. We used the vertical trajectory of the distal toe marker to quantify toe position. Previous studies of toe clearance and fall risk in the healthy elderly investigated toe clearance in mid-swing [26], and late swing. [28] However, close investigation of paretic leg displacement throughout swing reveals an atypical kinematic pattern distinct from both healthy controls and individuals with drop foot due to peroneal nerve injury. [29] The kinematic pattern noted post-stroke suggests early swing may be the relevant period of investigation. [29] Further, upon analysis of our data, we noted the critical toe clearance, identified by a local minimum of the vertical trajectory of the toe, in mid-swing, [26], [27], [30], [31] is absent in healthy controls when walking at speeds matched to our participants post-stroke. Similarly, this critical toe clearance characteristic in mid-swing was not systematically identifiable in our participants post-stroke. For these reasons, we identified minimal toe clearance in early swing, rather than the more common investigation of critical toe clearance in mid-swing. We defined minimal toe clearance (TC_min_) as the lowest vertical position of the trajectory of the toe marker during swing.

Shortening of the swing limb, rather than an absolute measure of clearance, provides a direct measure of the capacity for limb shortening to enable the swing limb to advance in front of the body without foot-floor contact. [30] Normalized limb length was calculated as the instantaneous hip-toe distance (HT_distance_) divided by the instantaneous vertical distance from the hip joint center to the floor (HF_distance_). [30] Limb shortening was quantified as the percent reduction in normalized limb length relative to the instantaneous height of the hip joint center. To quantify the capacity for limb shortening, we defined maximal limb shortening (LS_max_) as the highest percent reduction in normalized limb length during swing. Normalized limb length values less than 1 indicate limb shortening.

### Biomechanical Model

**Figure 1:**
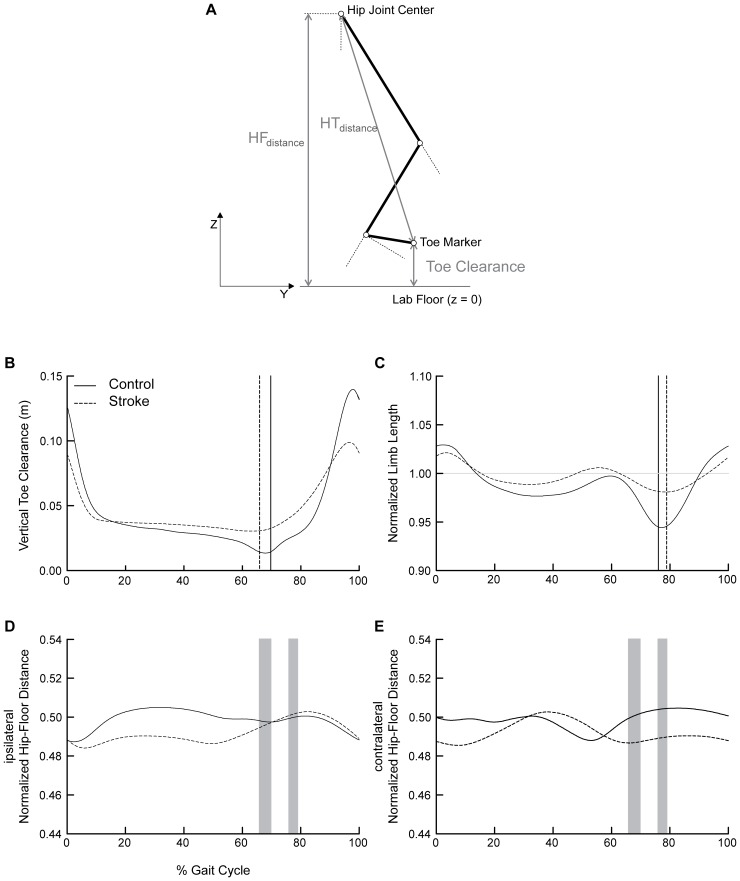
Gait events and hip-floor height. (a) Toe clearance served as our indicator of limb clearance. We used the trajectory of the distal toe marker to quantify toe position. Minimal toe clearance (TC_min_) was defined as the lowest vertical position of the trajectory of the toe marker during swing. Normalized limb length was calculated as the instantaneous hip-toe distance (HT_distance_) divided by the instantaneous vertical distance from the hip joint center to the floor (HF_distance_). [30] Limb shortening was quantified as the percent reduction in normalized limb length relative to the instantaneous height of the hip joint center. Maximal limb shortening (LS_max_) was defined as the highest percent reduction in normalized limb length during swing. (b) The vertical trajectory of the great toe marker, time normalized to the gait cycle. (c) Normalized limb length quantified as the hip-toe distance divided by the hip-floor distance. Values less than 1 indicate limb shortening. (d) Ipsilateral and (e) contralateral normalized hip-floor height quantified as the hip-floor height divided by the participant’s height. Controls are depicted in solid black, with participants post-stroke depicted with dashed lines in all panels. Vertical cursor lines represent: minimal toe clearance (b) and maximal limb shortening (c) for each group. Grey shaded regions (d and e) represent the range (across groups) of timing for minimal toe clearance (1^st^) and maximal limb shortening (2^nd^).

A planar model of the leg was used to investigate the relative contributions of sagittal plane joint angles to toe clearance and limb shortening. [30] By convention the model reports hip flexion, knee flexion, and ankle dorsiflexion angles as positive joint rotations (Figure 1). The model and sensitivity equations used in the current analysis were developed by Moosabhoy and Gard and are briefly described below. [30].

Vertical toe position (i.e., toe height) is a function of: i) vertical hip position, ii) thigh, shank, and foot segment lengths and iii) hip, knee, and ankle angles (*see Equation 2*). [30] As such, the relative contribution of each joint can be determined by calculating the partial derivative of the vertical toe position with respect to each joint angle (*see Equations 6–8*). [30].

The hip-toe distance is calculated via the Pythagorean theorem using the vertical and fore-aft coordinates of the hip joint center and distal toe marker (*see Equation 15*). [30] Again, the partial derivative with respect to the contributing joints (i.e., knee and ankle) quantifies the relative contribution of the knee and ankle to limb shortening (*see Equations 16–17*). [30].

### Outcome Measures

Hip flexion, knee flexion, and ankle dorsiflexion were assessed at two events of interest during the swing phase of gait: minimum toe clearance (TC_min_) and maximal limb shortening (LS_max_). The gait events TC_min_ and LS_max_ were identified and their timing, relative to the gait cycle, analyzed (Figure 1, panels b–c). To account for potential abnormal stance limb (e.g., vaulting) or pelvic motion (e.g., ipsilateral hip hiking) that could contribute to limb advancement, we quantified bilateral hip-floor heights, normalized to the participant’s height, at TC_min_ and LS_max_.

We used sensitivity analysis to determine the relative contribution of respective joint angles on toe clearance and limb shortening. [30] Sensitivity analysis quantifies the relationship between known inputs (i.e., swing limb sagittal plane joint motions) and an outcome of interest (e.g., paretic toe clearance) by calculating the partial derivative of the outcome with respect to each of the contributing inputs. [30] We assessed toe clearance sensitivity (TC_sensitivity_) at TC_min_ to investigate the relative contribution of hip flexion, knee flexion, and ankle dorsiflexion at this critical gait event. Thus, TC_sensitivity_ is defined as the partial derivative of toe clearance with respect to the sagittal plane hip, knee, and ankle joint motions. [30] Positive values of TC_sensitivity_ indicate that a positive rotation at a given joint (i.e., hip flexion, knee flexion, and ankle dorsiflexion) increases toe clearance, while negative sensitivities indicate positive sagittal plane motion decreases toe clearance. Further, we assessed limb shortening sensitivity (LS_sensitivity_) at LS_max_ to investigate the relative contribution of knee flexion and ankle dorsiflexion. LS_sensitivity_ is defined as the partial derivative of the normalized limb length with respect to knee and ankle flexion. Negative values of LS_sensitivity_ indicate a positive rotation at a given joint decreases the hip-toe distance, effectively shortening the limb. We also determined the timing of sensitivity peaks to assess differences in temporal coordination between groups.

Interpretation of sensitivity values requires simultaneous knowledge of the direction of joint motion. For example, in the second half of swing, TC_sensitivity_ with respect to knee flexion is negative suggesting knee flexion reduces toe clearance. However, the knee is extending during this time and thus motion at the knee increases toe clearance towards the end of swing. Accordingly, we quantified the estimated joint influence on toe clearance and limb shortening throughout the gait cycle by calculating the product of the sensitivity values and the point-to-point changes in the sagittal plane joint angles. It follows that a positive value of influence on toe clearance increases toe clearance whereas a negative influence value on limb shortening decreases the normalized limb length, and thus increases limb shortening.

### Statistical Analysis

A Student’s *t-*test was used to confirm speed matching between non-disabled and stroke participants. To test our first hypothesis, Student’s *t*-tests were used to test for group differences in: i) TC_min_ and LS_max_ during swing, and ii) their corresponding timing relative to the gait cycle. To test our second hypothesis, separate mixed-design ANOVAs were used to analyze Group × Joint data for: i) sagittal plane joint angles (2×3), ii) TC_sensitivity_ and joint influence on toe clearance at TC_min_ (2×3), and iii) LS_sensitivity_ and joint influence on limb shortening at LS_max_ (2×2) for the paretic and non-paretic limbs. To investigate stance leg and pelvic contributions, Student’s *t-*tests were used to test for group differences in normalized hip-floor heights. To control for Type I error, statistical significance was established at *p*<0.01 to ensure >99% likelihood that means actually differ. Tukey’s HSD post-hoc analyses were performed to isolate differences when significant main effects or interactions were detected. Statistical significance for interactions was noted at *p*<0.05. All statistical tests were performed with JMP software (version 9.0.2, SAS Institute Inc., Cary, NC).

## Results

Gait speed matching was confirmed between non-disabled (0.54±0.19 m/s) and post-stroke participants (0.54±0.26 m/s; *p* = 0.98). Similarly, cadence did not differ statistically between participants post-stroke (74.6±20.4 steps/min) and controls (59.3±15.0 steps/min; *p* = 0.02). Tables 1 through 3 present all statistical results for the outcomes relevant to TC_min_ and LS_max_.

**Table 1:** Timing and magnitude of gait events.

	Outcome	Control	Stroke	Overall test	Sig	Cohen’s d
**Paretic**	Toe-off timing (% GC)	68.94 (4.07)	63.97 (7.05)	*t*_(30)_ = −2.44	*p* = 0.02	0.86
	Minimum toe clearance (cm)	1.48 (0.69)	3.25 (0.34)	*t*_(30)_ = 9.17	*p*<0.0001*	3.25
	Minimum toe clearance timing (% GC)	69.74 (3.48)	65.9 (5.19)	*t*_(30)_ = −2.46	*p* = 0.02	0.87
	Limb shortening (%)	4.97 (0.59)	1.08 (1.07)	*t*_(30)_ = 12.73	*p*<0.0001*	4.51
	Limb shortening timing (%)	76.04 (0.98)	78.94 (1.01)	*t*_(30)_ = 8.20	*p*<0.0001*	2.91
	Ipsilateral normalized hip-floor height at TC_min_	0.50 (0.02)	0.50 (0.03)	*t*_(30)_ = 0.15	*p* = 0.88	0
	Contralateral normalized hip-floor height at TC_min_	0.50 (0.02)	0.49 (0.02)	*t*_(30)_ = −1.82	*p* = 0.08	0.5
	Ipsilateral normalized hip-floor height at LS_max_	0.50 (0.02)	0.50 (0.02)	*t*_(30)_ = 0.27	*p* = 0.79	0
	Contralateral normalized hip-floor height at LS_max_	0.50 (0.02)	0.49 (0.02)	*t*_(30)_ = −1.76	*p* = 0.09	0.5
**Nonparetic**	Toe-off timing (% GC)	69.21 (4.30)	74.51 (6.31)	*t*_(30)_ = 2.77	*p* = 0.009*	0.98
	Minimum toe clearance (cm)	1.26 (0.66)	2.98 (0.61)	*t*_(30)_ = 7.65	*p*<0.0001*	2.71
	Minimum toe clearance timing (% GC)	70.26 (3.62)	76.6 (4.55)	*t*_(30)_ = 4.36	*p*<0.0001*	1.54
	Limb shortening (%)	4.21 (0.79)	3.28 (0.90)	*t*_(30)_ = 3.07	*p* = 0.0045	1.10
	Limb shortening timing (%)	76.62 (1.11)	84.59 (1.33)	*t*_(30)_ = 18.41	*p*<0.0001*	6.51
	Ipsilateral normalized hip-floor height at TC_min_	0.50 (0.02)	0.48 (0.02)	*t*_(30)_ = −2.25	*p* = 0.03	1
	Contralateral normalized hip-floor height at TC_min_	0.51 (0.02)	0.49 (0.02)	*t*_(30)_ = −2.65	*p* = 0.01	1
	Ipsilateral normalized hip-floor height at LS_max_	0.50 (0.02)	0.48 (0.02)	*t*_(30)_ = −2.51	*p* = 0.02	1
	Contralateral normalized hip-floor height at LS_max_	0.51 (0.02)	0.49 (0.02)	*t*_(30)_ = −2.86	*p* = 0.008*	1

* significant difference between groups.

Data are mean (sd). Control values reflect the limb against which the stroke limb was tested (i.e., the right limb was designated for comparison against the paretic limb). Note: i) exaggerated paretic toe clearance post-stroke despite limited limb shortening, ii) lack of differences in the normalized hip-floor heights at minimal toe clearance, bilaterally.

**Table 2:** Relative contributions to toe clearance.

	Outcome	Joint	Control	Stroke	Overall test	Sig	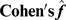
**Paretic**	Peak TC sensitivity timing (% GC)	Hip†	67.48 (1.07)	63.28 (2.27)	*F*_(2,30)_ = 27.57	*p*<0.0001†	0.74
		Knee†	70.9 (0.81)	67.36 (2.39)			
		Ankle†	70.17 (0.79)	68.81 (2.01)			
	Angle at TC_min_ (degrees)	Hip	16.87 (10.12)	22.59 (7.51)	*F*_(2,30)_ = 21.51	*p*<0.0001†	0.65
		Knee†	48.49 (12.54)	25.62 (15.29)			
		Ankle	−2.61 (3.62)	−5.19 (7.30)			
	TC sensitivity at TC_min_ (m/deg)	Hip†	−0.04 (0.10)	0.22 (0.10)	*F*_(2,30)_ = 59.39	*p*<0.0001†	1.10
		Knee†	0.16 (0.07)	−0.09 (0.11)			
		Ankle	0.05 (0.02)	0.11 (0.02)			
	Influence of TC sensitivity (m)	Hip†	−0.004 (0.01)	0.01 (0.01)	*F*_(2,30)_ = 15.40	*p*<0.0001†	0.55
		Knee†	0.02 (0.02)	0.004 (0.01)			
		Ankle	0.002 (0.003)	−0.001 (0.003)			
**Nonparetic**	Peak TC sensitivity timing (% GC)	Hip†	67.83 (0.47)	69.08 (1.76)	*F*_(2,30)_ = 93.32	*p*<0.0001†	1.39
		Knee†	71.33 (0.70)	76.73 (0.83)			
		Ankle†	70.44 (0.62)	76.34 (0.69)			
	Angle at TC_min_ (degrees)	Hip†	14.19 (5.94)	35.60 (12.69)	*F*_(2,30)_ = 18.15	*p*<0.0001†	0.60
		Knee	45.94 (11.70)	50.00 (12.19)			
		Ankle	−2.98 (2.97)	−2.13 (5.07)			
	TC sensitivity at TC_min_ (m/deg)	Hip†	−0.06 (0.06)	0.19 (0.11)	*F*_(2,30)_ = 59.12	*p*<0.0001†	1.10
		Knee†	0.16 (0.07)	0.01 (0.06)			
		Ankle	0.05 (0.02)	0.10 (0.02)			
	Influence of TC sensitivity (m)	Hip†	−0.01 (0.01)	0.03 (0.02)	*F*_(2,30)_ = 38.68	*p*<0.0001†	0.89
		Knee†	0.03 (0.02)	0.01 (0.02)			
		Ankle	0.002 (0.003)	−0.002 (0.01)			

Cohen's 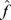 effect size for factorial ANOVA.

† significant statistical interaction (group × joint) and the location of effect revealed upon post-hoc testing.

TC: toe clearance.

TC_min_: minimal toe clearance.

Data are mean (sd). Control values reflect the limb against which the stroke limb was tested (i.e., the right limb was designated for comparison against the paretic limb). Paretic knee flexion is limited at minimal toe clearance, whereas nonparetic hip flexion is exaggerated. Individual joint contributions to toe clearance are reversed in both limbs post-stroke such that the hip and knee are increased and reduced, respectively.

**Table 3:** Relative contributions to limb shortening.

	Outcome	Joint	Control	Stroke	Overall test	Sig	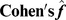
**Paretic**	Peak LS sensitivity timing (% GC)	Knee†	74.81 (0.86)	72.41 (3.28)	*F*_(1,30)_ = 604.84	*p*<0.0001†	2.51
		Ankle†	71.83 (0.86)	74.74 (3.18)			
	Angle at LS_max_ (degrees)	Hip	22.94 (11.43)	25.60 (8.92)	*F*_(2,30)_ = 24.30	*p*<0.0001†	0.70
		Knee†	50.02 (10.73)	23.90 (14.33)			
		Ankle	−0.25 (2.93)	−5.42 (6.90)			
	LS sensitivity at LS_max_ (m/deg)	Knee†	−0.14 (0.04)	−0.04 (0.06)	*F*_(1,30)_ = 34.29	*p*<0.0001†	0.59
		Ankle	−0.06 (0.01)	−0.09 (0.02)			
	Influence of LS sensitivity (m)	Knee	0.01 (0.02)	0.003 (0.01)	*F*_(1,30)_ = 0.65	*p* = 0.43	
		Ankle	−0.002 (0.003)	0.0005 (0.002)			
**Nonparetic**	Peak LS sensitivity timing (% GC)	Knee†	75.76 (1.74)	83.64 (1.62)	*F*_(1,30)_ = 17.97	*p* = 0.0002†	0.42
		Ankle†	73.05 (2.43)	82.44 (2.45)			
	Angle at LS_max_ (degrees)	Hip†	22.57 (7.18)	36.88 (12.43)	*F*_(2,30)_ = 13.96	*p*<0.0001†	0.52
		Knee	49.68 (8.68)	49.90 (11.32)			
		Ankle	−0.63 (2.39)	−2.21 (4.99)			
	LS sensitivity at LS_max_ (m/deg)	Knee	−0.14 (0.03)	−0.13 (0.04)	*F*_(1,30)_ = 1.28	*p* = 0.27	
		Ankle	−0.06 (0.01)	−0.07 (0.02)			
	Influence of LS sensitivity (m)	Knee	0.01 (0.02)	0.01 (0.03)	*F*_(1,30)_ = 0.01	*p* = 0.91	
		Ankle	−0.001 (0.003)	0.002 (0.01)			

Cohen’s 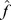 effect size for factorial ANOVA.

† significant statistical interaction (group × joint) and the location of effect revealed upon post-hoc testing.

LS: limb shortening.

LS_max_: maximal limb shortening.

Data are mean (sd). Control values reflect the limb against which the stroke limb was tested (i.e., the right limb was designated for comparison against the paretic limb). Note paretic knee flexion and its contribution to limb shortening are limited post-stroke. Nonparetic hip flexion is exaggerated post-stroke.

### Minimum Toe Clearance in Swing

#### Toe clearance magnitude and timing

At TC_min_, the vertical displacement of the paretic limb toe marker was higher in participants post-stroke (3.25±0.34 cm) than healthy controls (1.48±0.69 cm; *p*<0.0001; Figure 1). The timing of minimum toe clearance relative to the gait cycle (GC) did not differ significantly between participants post-stroke (65.9±5.19%GC) and non-disabled controls (69.74±3.48%GC; *p* = 0.02).

#### Normalized hip-floor height at minimal toe clearance

No differences were detected in the ipsilateral and contralateral normalized hip-floor heights, at TC_min_, between participants post-stroke and controls (Figure 1 d–e). This was consistent for both paretic (*p* = 0.88 and *p* = 0.08, respectively) and nonparetic (*p* = 0.03 and *p* = 0.01, respectively) gait cycles.

#### Sagittal plane joint angles at minimal toe clearance

**Figure 2:**
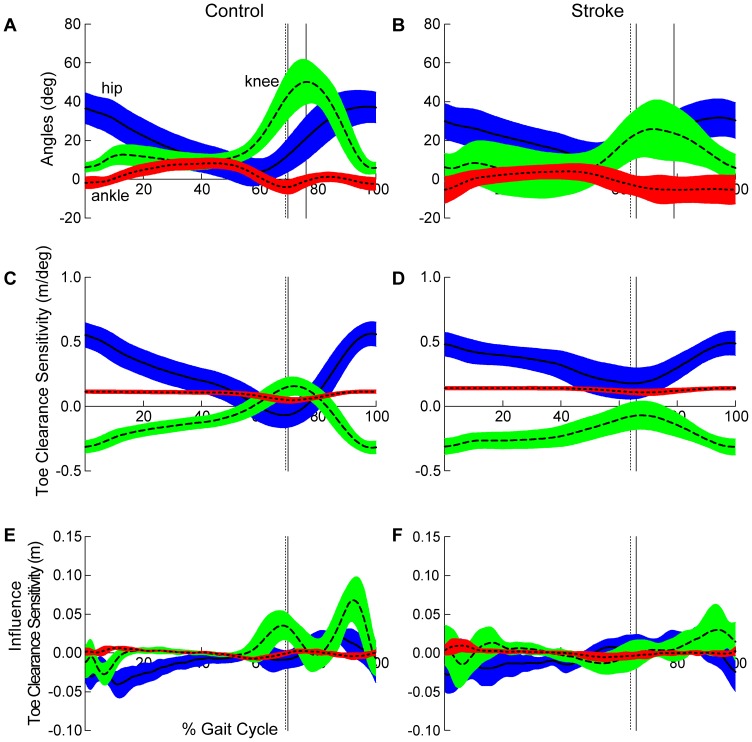
Sagittal plane joint angles and contributions to paretic toe clearance. Healthy controls are depicted in the left and participants post-stroke in the right column. (a and b) Sagittal plane joint angles. Vertical cursor lines in represent toe off (dashed), minimal toe clearance (1^st^ solid), and maximal limb shortening (2^nd^ solid). (c and d) Toe clearance sensitivity - defined as the partial derivative of toe clearance with respect to sagittal plane hip, knee, and ankle angles. Positive values of TC_sensitivity_ indicate a positive rotation at a given joint increases toe clearance. Note that the ankle contribution (dotted line) to toe clearance is approximately equal in control and stroke. However, the timing of sensitivity peaks differ between groups (Table 1). (e and f) Estimated influence of toe clearance sensitivity quantifies the contribution of each joint to toe clearance, regardless of direction of joint motion. Note the pattern of joint influence in healthy controls clearly indicating the knee serves as the primary contributor to toe clearance. Vertical cursors in (c–f) represent toe off (dashed) and minimal toe clearance (solid) for each group. Hip, knee, and ankle curves are depicted as solid, dashed, and dotted lines, respectively. Error clouds denote ±1 standard deviation. All curves are time normalized to the gait cycle.

A significant statistical interaction revealed differences in paretic limb sagittal plane joint angles between controls and participants post-stroke at TC_min_ (Group × Joint; *p*<0.0001). Post-hoc testing revealed no differences between groups for hip flexion or ankle dorsiflexion; however, knee flexion was lower following stroke (25.62±15.29°) compared to controls walking at matched speeds (48.49±12.54°; Figure 2). Similarly, a significant interaction was detected in the non-paretic limb (Group × Joint; *p*<0.0001). Post-hoc testing identified non-paretic limb hip flexion (35.60±12.69°) was greater than controls (14.19±5.94°).

#### Toe clearance sensitivity at minimal toe clearance

A significant statistical interaction detected differences in the pattern of paretic TC_sensitivity_ at TC_min_ between controls and participants post-stroke (Group × Joint; *p*<0.0001; Figure 2). Again, post-hoc analysis revealed no differences in TC_sensitivity_ with respect to ankle dorsiflexion (Control: 0.05±0.02 m/deg; Stroke: 0.11±0.02 m/deg). However, the pattern of TC_sensitivity_ between groups with respect to hip and knee flexion was reversed. Following stroke, TC_sensitivity_ with respect to hip flexion was greater than controls (Control: −0.04±0.10 m/deg; Stroke: 0.22±0.10 m/deg) indicating hip flexion provided a greater relative contribution to toe clearance while TC_sensitivity_ with respect to knee flexion was less than controls (Control: 0.16±0.07 m/deg; Stroke: −0.09±0.11 m/deg). In participants post-stroke, TC_sensitivity_ with respect to knee flexion remained negative throughout the entire gait cycle indicating knee flexion decreases toe clearance at the point of minimal toe clearance. The nonparetic limb (Group × Joint; *p*<0.0001) revealed a similar reversal in the pattern between the hip (Control: −0.06±0.06 m/deg; Stroke: 0.19±0.11 m/deg) and knee (Control: 0.16±0.07 m/deg; Stroke: 0.01±0.06 m/deg) contributions to toe clearance.

#### Estimated joint influence on toe clearance

A significant statistical interaction identified differences in joint influence on toe clearance between participants post-stroke and controls, bilaterally (Group × Joint; *p*’s <0.0001). Regardless of direction of joint motion, following stroke the knee joint contribution to toe clearance was less than controls, whereas the hip joint contribution was exaggerated, in both the paretic (Control: hip: −0.004±0.10 m, knee: 0.02±0.02 m; Stroke: hip: 0.01±0.01 m, knee: 0.004±0.01 m) and nonparetic (Control: hip: −0.01±0.01 m, knee: 0.03±0.02 m; Stroke: hip: 0.03±0.02 m, knee: 0.01±0.02 m) limbs (Figure 2).

### Maximum Limb Shortening in Swing

#### Limb shortening magnitude and timing

Paretic limb shortening at LS_max_ was less post-stroke (1.07±1.07%) relative to controls (4.97±0.59%; *p*<0.0001). The timing of maximal limb shortening occurred later in the gait cycle for participants post-stroke (78.94±1.01%GC) than for controls (76.04±0.98%GC; *p*<0.0001).

#### Normalized hip-floor height at maximal limb shortening

No differences between post-stroke and control groups were detected in the ipsilateral and contralateral normalized hip-floor heights of the paretic gait cycle (*p* = 0.79 and *p* = 0.09, respectively; Figure 1 d–e). However, post-stroke the contralateral normalized hip-floor height at LS_max_ was reduced during the nonparetic gait cycle (*p* = 0.008; i.e., paretic normalized hip-floor height during paretic single support).

#### Sagittal plane joint angles at maximal limb shortening

A significant statistical interaction detected differences between groups in both paretic and nonparetic (Group × Joint; *p*’s <0.0001) limb sagittal plane joint angles at LS_max_. Similar to TC_min_, post-hoc analysis revealed no differences in paretic hip flexion and ankle dorsiflexion between groups at LS_max_. However, limited paretic knee flexion was noted following stroke (23.90±14.33°) compared to non-disabled controls (50.22±10.73°). In the nonparetic limb, post-hoc analysis revealed hip flexion was exaggerated following stroke (36.88±12.43°) compared to controls (22.57±7.18°).

#### Limb shortening sensitivity at maximal limb shortening

**Figure 3:**
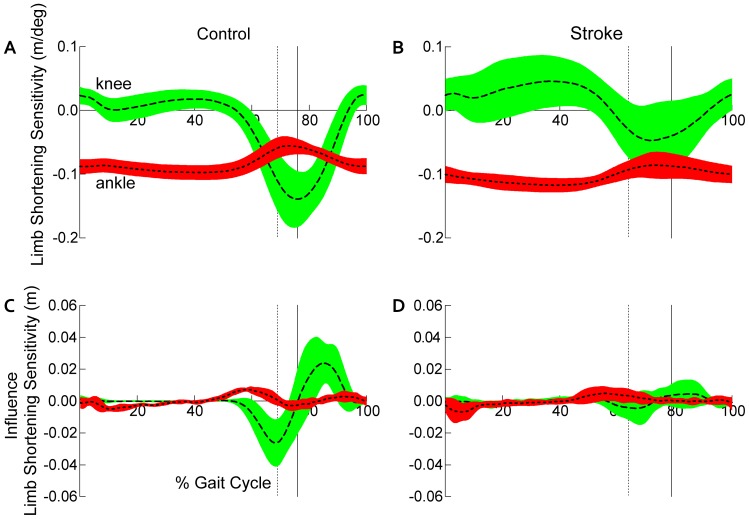
Contributions to paretic limb shortening. Limb shortening sensitivity, normalized to the gait cycle, for (a) healthy controls and (b) participants post-stroke. Limb shortening sensitivity - defined as the partial derivative of limb shortening with respect to sagittal plane knee and ankle angles. Negative values of limb shortening sensitivity indicate a positive rotation at a given joint decreases the normalized limb length, thus increasing limb shortening. Note, the timing of sensitivity peaks differs between groups (Table 1). To eliminate the direction of joint motion from the interpretation of each joint’s contribution to limb shortening, we illustrate the estimated influence of each joint, normalized to the gait cycle, for (c) healthy controls and (d) participants post-stroke. Vertical cursors represent toe off (dashed) and maximal limb shortening (solid). Knee and ankle curves are depicted as dashed and dotted lines, respectively. Error clouds denote ±1 standard deviation.

Consistent with results reported for joint angles, a significant statistical interaction detected differences in the pattern of paretic LS_sensitivity_ at LS_max_ between groups (Group × Joint; *p*<0.0001). No differences were detected in the contribution of ankle dorsiflexion to limb shortening between controls and participants post-stroke. However, participants post-stroke (−0.04±0.06 m/deg) revealed a lower magnitude of LS_sensitivity_ with respect to knee flexion than controls (−0.14±0.04 m/deg; Figure 3). Given the negative sign of the sensitivity values for both groups, the lower magnitude indicates that knee flexion is less influential to paretic limb shortening post-stroke than for controls. While no Group × Joint interaction was detected in the nonparetic limb (*p* = 0.27), a main effect of Joint (*F*_(1,30)_ = 67.04, *p*<0.0001) was revealed indicating that knee flexion provides a greater contribution to limb shortening than the ankle in both non-disabled controls and the nonparetic limb of persons post-stroke.

#### Estimated joint influence on limb shortening

In contrast to the joint influence on toe clearance, no significant differences were detected between participants post-stroke and controls in the pattern of joint influence on limb shortening, when evaluated at the point of maximal limb shortening (Group × Joint; paretic: *p* = 0.43, nonparetic: *p* = 0.91; Figure 3). However, visual inspection of the joint influence curves (Figure 3) illustrates differences in both the magnitude and timing of knee influence on limb shortening. Importantly, the knee influence on limb shortening precedes LS_max_ in controls whereas this influence appears extremely limited and ill-timed post-stroke.

## Discussion

### Debunking the Notion of So-called Foot Drop Post-Stroke

To our knowledge this is the first study to quantify the relationship between changes in hip, knee, and ankle kinematics and toe clearance and limb shortening post-stroke and thus advances understanding regarding the mechanisms responsible for impaired paretic limb advancement. Our current findings fail to evince foot drop (i.e., impaired dorsiflexor function) as a cause of insufficient toe clearance post-stroke and point to other underlying mechanisms of impaired paretic limb advancement. Our failure to confirm the phenomenon of so-called foot drop in persons post-stroke motivates further research using quantitative approaches to identify the actual underlying cause(s) of hemiparetic gait dysfunction. We recommend such approaches to inform development of appropriately targeted interventions, which will have a higher likelihood of producing meaningful outcomes for persons post-stroke. A productive first step is adoption of appropriate terminology – impaired limb advancement – which incorporates the multifactorial nature of hemiparetic gait dysfunction and thus motivates redirection of stroke rehabilitation to target remediation of all contributing factors.

Contrary to commonly held beliefs, [4] our findings reveal the contribution of ankle dorsiflexion to toe clearance and limb shortening does not differ between individuals post-stroke and healthy, non-disabled individuals walking at matched speeds. Indeed, rather than dorsiflexion dysfunction, our data reveal a prominent disruption in the phasic interdependence of the hip and knee. These findings are consistent across multiple variables and strongly implicate dysfunction at the knee, as noted by reduced knee flexion during swing phase, as a fundamental component of impaired paretic limb advancement. Failure to identify deficient ankle dorsiflexion leaves the paretic limb hip flexors to compensate for inadequate knee flexion in attaining sufficient limb shortening for clearance and advancement during swing phase.

### Lack of Evidence for Dorsiflexor Impairment Post-Stroke

Neither EMG nor kinematic findings reported in the literature support the commonly held premise of dorsiflexor impairment post-stroke. [10]–[14] Rather than the deficient pattern of so-called foot drop typically argued as the rationale for targeting dorsiflexor dysfunction for intervention and/or orthotic management, [4] multiple authors report prolonged [11] or normal [12] activation of tibialis anterior during swing and normal co-activation between tibialis anterior and medial gastrocnemius muscles. [13] Furthermore, several authors report similarity between persons post-stroke and controls in the ankle angle excursions during swing. [10], [14] The ankle angles we observed post-stroke do not reveal kinematic profiles of genuine foot drop as illustrated by experimentally induced nerve block to the common peroneal nerve. [8] Importantly, some authors who argue for the prominence of impaired dorsiflexion post-stroke acknowledge that impaired limb advancement results from multiple factors and cannot be isolated to deficient dorsiflexor muscle activity. [12], [14] The lack of difference between groups in ankle angles at either critical gait event, TC_sensitivity_ to ankle dorsiflexion at TC_min_, and LS_sensitivity_ to ankle dorsiflexion at LS_max_ argue against dorsiflexor dysfunction as the fundamental impairment of limb advancement in persons post-stroke.

### Appropriate Identification of Impairments

Moosabhoy and Gard explain that floor clearance and limb shortening are served by a, “‘phasic interdependence’ that enables and requires the rotation at one joint to complement those at the other joints in achieving the desired objectives of shortening the leg while advancing it in front of the body”. [30] Taken together, our observations of: reversed TC_sensitivity_ to hip and knee flexion between groups at TC_min_, limited knee flexion in swing post-stroke, and limited contribution of knee flexion to limb shortening following stroke, are consistent with disruption of this phasic interdependence between the hip and the knee. Close biomechanical coupling between the hip and knee is recognized in healthy individuals. [32] Exaggerated hip flexion contribution to toe clearance may be a compensatory manifestation of this coupling.

Given the dynamic role of the plantarflexors in increasing the rate of knee flexion during pre-swing, [33] our findings are consistent with plantarflexor, rather than dorsiflexor, dysfunction as an underlying impairment of limb advancement. There is considerable evidence that plantarflexor impairment plays an important role in paretic limb advancement.[14], [25], [34]–[37] Moreover, interventions utilizing novel stimulation parameters [38] and robotic devices [39] targeting both the dorsiflexors and plantarflexors have shown promising results at improving paretic ankle motor control, paretic single limb support, gait kinematics and kinetics, and overground walking speed. Good response to walking-related intervention post-stroke is associated with improvements in peak ankle plantarflexor angle and power production in pre-swing. [3] To confirm the role of plantarflexor dysfunction in limb advancement, future studies incorporating kinetic and EMG analyses are needed.

Exaggerated biomechanical patterns (i.e., kinematics, kinetics and EMG) are frequently observed in the nonparetic limb and it is argued these are a manifestation of compensatory mechanisms to maintain walking function post-stroke. [10], [35], [37] Our findings of limited paretic limb knee flexion and exaggerated nonparetic hip flexion during swing concur with this body of previous work and emphasize the presence of bilateral involvement following stroke.

### Biomechanical Model

We used a unilateral planar model to investigate the influence of swing limb joint motion on paretic limb clearance and shortening. This model was sufficient to answer our primary question regarding the relative contribution of ankle dorsiflexion on paretic limb clearance and allowed identification of other contributing mechanisms to impaired limb advancement following stroke. We attempted to account for abnormal stance limb (e.g., vaulting) and pelvic motion (e.g., ipsilateral hip hiking) that may contribute to paretic limb clearance by quantifying the ipsilateral and contralateral hip-floor heights at the critical gait events. We did not detect any differences between groups suggesting the increased toe clearance seen post-stroke could not be attributed to either of these compensatory patterns. Still, we have not assessed contributions throughout the stance limb, and specific to the pelvis. Winter has described the link segment contributing to swing limb foot clearance to include the stance foot up to the hip, across the pelvis, and down to the distal end of the swing foot. [31] Using this bilateral model, Winter illustrated that swing limb foot clearance is most sensitive to stance limb hip abduction in healthy individuals. [31] The known biomechanical dysfunction at the pelvis and disruptive influence of the nonparetic limb following stroke motivates further investigation to determine stance limb and pelvic contributions to paretic limb clearance.[40]–[45] A bilateral biomechanical model would provide a more comprehensive picture of factors that contribute to paretic limb clearance and limb shortening.

### Timing of Gait Events

Our results differ from those of Moosabhoy and Gard who found the relative contributions to toe clearance were greatest from ankle dorsiflexion, then hip flexion and least from knee flexion. [30] Similarly, they identified the greatest contribution to limb shortening to be from ankle dorsiflexion. [30] However, there is an important methodological distinction to be made between the two studies. Moosabhoy and Gard investigated the relative contributions to toe clearance and limb shortening at a point of *critical* toe clearance, identified by a ‘local minimum’ of the vertical trajectory of the toe which occurs approximately half way through swing. [30] However, inspection of vertical toe clearance in our study (Figure 1b) reveals a more subtle inflection point in controls walking at slow speeds, and the absence of this local minimum in the trajectory of toe clearance post-stroke. Further, investigation of a clinical population in which foot drop is suspect, suggests quantifying the relative contributions to *minimal* toe clearance would be most relevant. [29] We note the timing of minimal toe clearance post-stroke is immediately following toe off, which is consistent with clinical observation of impaired limb clearance in this population. [29] We also identified the point of maximal limb shortening to be the relevant time of investigation for the relative contributions to limb shortening, given the impairment of limb shortening following stroke.

We also expanded the analysis previously presented [30] by quantifying the influence of each joint on toe clearance (Figure 2e) and limb shortening (Figure 3c), regardless of the direction of joint motion. We note the knee is primarily responsible for both of these tasks in controls, even at slow walking speeds. However, distinctly different patterns are revealed post-stroke (Figures 2f and 3d) with the influence of the knee markedly reduced.

## Conclusions/Implications

Ankle angles and the ankle dorsiflexion contribution to toe clearance and limb shortening are similar between non-disabled and post-stroke groups. Considered in combination with differences in the hip and knee contributions to toe clearance and knee contributions to limb shortening, these similarities argue strongly against dorsiflexor dysfunction as the fundamental impairment of limb advancement post-stroke. Marked reversal of the roles of hip and knee flexion contributing to toe clearance points to disruption of dynamic inter-joint coordination, which most likely results from impairment of the dynamic contribution to knee flexion by the plantarflexors in preparation for swing. These findings motivate reconsideration of the notion that foot drop contributes significantly to gait dysfunction post-stroke. Accordingly, redirecting the focus of treatment for hemiparetic walking dysfunction to the contributory neuromechanical impairments identified through quantitative biomechanical analyses will improve both the efficacy and outcomes of rehabilitation interventions and reduce stroke-related physical disability and health-related costs.
